# Letter to the Editor: Leveraging artificial intelligence to improve patient education for hypertensive disorders of pregnancy

**DOI:** 10.1002/pmf2.70104

**Published:** 2025-09-17

**Authors:** Amos Grünebaum

**Affiliations:** ^1^ Northwell Health Donald and Barbara Zucker School of Medicine at Hofstra/Northwell Hempstead New York USA

To the Editor

I congratulate the Society for Maternal‐Fetal Medicine's (SMFM) Special Statement on quality metrics for hypertensive disorders of pregnancy (HDP), which highlights a critical gap: inadequate patient education about lifelong cardiovascular risks following pregnancy complications [1]. We believe that while the proposed Metric 1 ensuring > 99% of HDP patients receive predischarge education represents important progress, the implementation of it faces significant barriers. We are writing this letter to recommend ways of implementing this goal.

Both ACOG and NIH have long recommended that patient education materials be written at 6th–8th grade reading levels [2, 3]. However, recent evidence demonstrates widespread failure to meet these standards [4]. Analysis of 134 ACOG patient education pamphlets revealed only 35.1% met plain language standards, with most requiring 10th–12th grade reading ability. Most concerning, readability has worsened over 25 years, with complexity increasing by 1.0–3.7 grade levels across multiple metrics [4]. This disconnect particularly affects populations with lower health literacy who already face higher risks for adverse outcomes. Supporting information 1.

We believe that artificial intelligence (AI) offers a practical solution to bridge this gap. A collaboration with a generative artificial intelligence mode such as Claude (Anthropic) makes it possible to develop a patient education handout addressing SMFM's specific recommendations while maintaining 6th–8th grade readability. It enables rapid iteration, incorporating critical elements such as specific blood pressure thresholds, realistic healthcare access options, and clear warning signs. The Appendix we developed serves as a template that can be easily modified for local population needs with appropriate prompt engineering while preserving evidence‐based accuracy and appropriate readability levels. The material achieved a Flesch Reading Ease score of 72.1 (7th grade level) and a Flesch–Kincaid Grade Level of 6.9, successfully meeting NIH recommendations for 8th grade readability and plain language standards. This represents a significant improvement over typical healthcare materials, where most ACOG pamphlets require 10th–12th grade reading ability. It maintains clinical accuracy while incorporating complex medical concepts in language accessible to patients with average health literacy skills.

AI can democratize high‐quality patient education development, enabling providers to create customized, guideline‐compliant materials efficiently. Given that Black patients and other marginalized populations experience disproportionately worse maternal health outcomes, ensuring equitable access to comprehensible health information becomes critical for reducing disparities. As we pursue the ambitious goal of > 99% compliance with predischarge education, we should embrace technologies that deliver better, more accessible patient care at scale. The attached handout demonstrates what is achievable when healthcare expertise combines with AI capabilities.

## CONFLICT OF INTEREST STATEMENT

The author declares no conflicts of interest.

## Supporting information

Supporting information

## References

[1] Society for Maternal‐Fetal Medicine (SMFM) . 2025. “Quality Metrics for Hypertensive Disorders of Pregnancy—Patient Education and Transition to Primary Care.” Pregnancy 1: e70023. 10.1002/pmf2.70023 PMC13344586 42596957

[2] Committee on Patient Safety and Quality Improvement , Committee on Health Care for Underserved Women . 2016. “Health Literacy to Promote Quality of Care: ACOG Committee Opinion No. 676.” Obstetrics and Gynecology 128(4): e183–6. 27661657 10.1097/AOG.0000000000001714

[3] National Cancer Institute . 2003. Clear & Simple: Developing Effective Print Materials for Low‐Literate Readers. Bethesda, MD: National Institutes of Health; NIH Publication No. 03‐5534.

[4] Grünebaum, A. , S. L. Pollet , R. McLeod‐Sordjan , G. Bachman , and F. A. Chervenak . 2025. “Patient Education Materials: Improving Readability to Advance Health Equity.” Journal of Perinatal Medicine. published online July 25, 2025. PMID: 40708234. 10.1515/jpm-2025-0368 40708234

