# Supplementary material for: Letter to the Editor: Leveraging artificial intelligence to improve patient education for hypertensive disorders of pregnancy
Source: Pregnancy (Hoboken). 2025 Sep 17;1(5):e70104. doi: 10.1002/pmf2.70104 (PMC13344566; doi:10.1002/pmf2.70104)
Supplement: Supplementary file 1 [file PMF2-1-e70104-s001.docx]

**Attachment: Patient Education Handout**

**High Blood Pressure During Pregnancy: Your Health for Life**

**What You Need to Know About Your Future Health**

**You had high blood pressure during pregnancy.** This is also called a "hypertensive disorder of pregnancy" or HDP. This may sound scary, but knowing about it helps you stay healthy for years to come.

**Your Blood Pressure After Pregnancy**

Your blood pressure will likely go back to normal in the days or weeks after your baby is born. This is good news! However, even when your blood pressure returns to normal, having high blood pressure during pregnancy means you have a higher chance of health problems later in life.

You may develop high blood pressure again at some point. This could happen months from now, years from now, or even decades later. This is why it's so important to keep track of your health and see your doctor regularly.

**Health Risks You Should Know About**

Having high blood pressure during pregnancy puts you at higher risk for serious health problems. These include heart problems like heart attacks, heart failure, and irregular heartbeats. You also have a higher chance of having a stroke, developing chronic high blood pressure, and getting kidney disease.

There's also something called metabolic syndrome. This is when you have several health problems together: high levels of bad cholesterol, high blood pressure, pre-diabetes, and extra weight around your middle. When these happen together, they make heart disease and diabetes more likely.

We're not trying to scare you. We want you to know so you can take steps to stay healthy and catch any problems early when they're easier to treat.

**What You Need to Do Now**

**Call Your Doctor or Clinic Today**

You need to make an appointment with a doctor who can help you with your ongoing health care. This could be your ObGyn, a family doctor, a clinic, or any healthcare provider who takes care of adults. These providers are often very busy and may not have appointments for several weeks, so call today. When you call, tell them you had high blood pressure during pregnancy. This is important information they need to know.

Bring this paper with you to your appointment and give your provider a copy. Ask your doctor or nurse to check you every year for the health problems we talked about.

**What Your Healthcare Provider Should Check Every Year**

Your healthcare provider should check your blood pressure, cholesterol levels, and blood sugar every year. These simple tests can catch problems early before they become serious.

**Monitor Your Blood Pressure at Home**

You need to get your blood pressure checked in the next few days. If you had severe high blood pressure, get it checked within 3-7 days. If your high blood pressure was not severe, get it checked within 7-10 days. Follow whatever plan your delivery team gave you for this check-up.

**Consider getting a home blood pressure machine.** You can buy one at most drug stores or online. Having one at home lets you keep track of your blood pressure between doctor visits. Check your blood pressure 2-3 times each day for the first few weeks after delivery. Also check it any time you have symptoms like headaches or feel unwell.

**When to call your doctor or nurse:** If your blood pressure readings are 150/100 or higher, call your healthcare provider right away. If your readings are 160/110 or higher, especially if you have symptoms like severe headache, vision changes, or upper belly pain, go to the emergency room immediately or call 911.

Write down your blood pressure numbers and bring them to your appointments. This information helps your doctors take better care of you.

**Warning Signs - Get Help Right Away**

**Call 911 or go to the emergency room if you have any of these symptoms:** a severe headache that won't go away, changes in your vision like blurriness or seeing spots or flashing lights, bad pain in your upper belly under your ribs, or trouble breathing or shortness of breath.

These symptoms could mean your blood pressure is dangerously high and you need help right away. Don't wait or try to tough it out. Get medical help immediately.

**Remember: You Are Not Alone**

Many women have high blood pressure during pregnancy. You are not the only one going through this. The most important thing is that you now know about your risks, which puts you ahead of many women who don't get this information.

Knowing your risks helps you stay healthy. When you see your healthcare provider regularly and get the right tests, you can prevent serious problems or catch them early when they're easier to treat. Early treatment works much better than waiting until problems get worse.

**Questions?**

**Contact your delivery hospital:** ________________

**Your healthcare provider or clinic:** ____________________

**Emergency: Call 911**

*Keep this paper with your important health records. Share it with all your doctors.*
